# Dietary Polyphenols against Oxidative Stress in Head and Neck Cancer: What's New, What's Next

**DOI:** 10.7150/jca.90545

**Published:** 2024-01-01

**Authors:** Andrea Ballini, Khrystyna Zhurakivska, Giuseppe Troiano, Lorenzo Lo Muzio, Vito Carlo Alberto Caponio, Francesca Spirito, Rosa Porro, Martina Rella, Stefania Cantore, Roberto Arrigoni, Mario Dioguardi

**Affiliations:** 1 Department of Clinical and Experimental Medicine, University of Foggia, Foggia, Italy.; 2 Department of Informatics, University of Bari “Aldo Moro”, Bari, Italy.; 3 AULSS4 - Veneto Orientale - Portogruaro, Venice, Italy.; 4 Department of Precision Medicine, University of Campania “Luigi Vanvitelli”, Naples, Italy.; 5 CNR Institute of Biomembranes, Bioenergetics and Molecular Biotechnologies (IBIOM), Bari, Italy.

**Keywords:** head and neck cancer, polyphenol, oxidative stress, cancer metabolism, reactive oxygen species (ROS), clinical biochemistry, translational medicine, polyphenol therapeutic potential, clinical studies

## Abstract

Head and neck cancers (HNC) are a worldwide health problem, accounting for over 5% of all types of cancers. Their varied nature makes it sometimes difficult to find clear explanations for the molecular mechanisms that underline their onset and development. While chemio- and radiotherapy are clearly not to be dismissed, we cannot undervalue the effect that polyphenols - especially dietary polyphenols - can have in helping us to cope with this medical emergency. By influencing several different proteins involved in numerous different metabolic pathways, polyphenols can have a broad spectrum of biological action and can hopefully act synergistically to tackle down head and neck cancer. Moreover, being natural molecules, polyphenols does not present any side effects and can even enhance drugs efficacy, making our clinical therapy against head and neck cancer more and more effective. Certainly, oxidative stress plays an important role, altering several molecular pathways, lowering the body's defenses, and ultimately helping to create a microenvironment conducive to the appearance and development of the tumor. In this regard, the regular and constant intake of foods rich in polyphenols can help counteract the onset of oxidative stress, improving the health of the general population. In this review, we highlight the role of polyphenols in managing oxidative stress, with such positive effects that they can be considered new tools to use in our anti-head and neck cancer strategy.

## 1. Introduction

**Figure 1:**
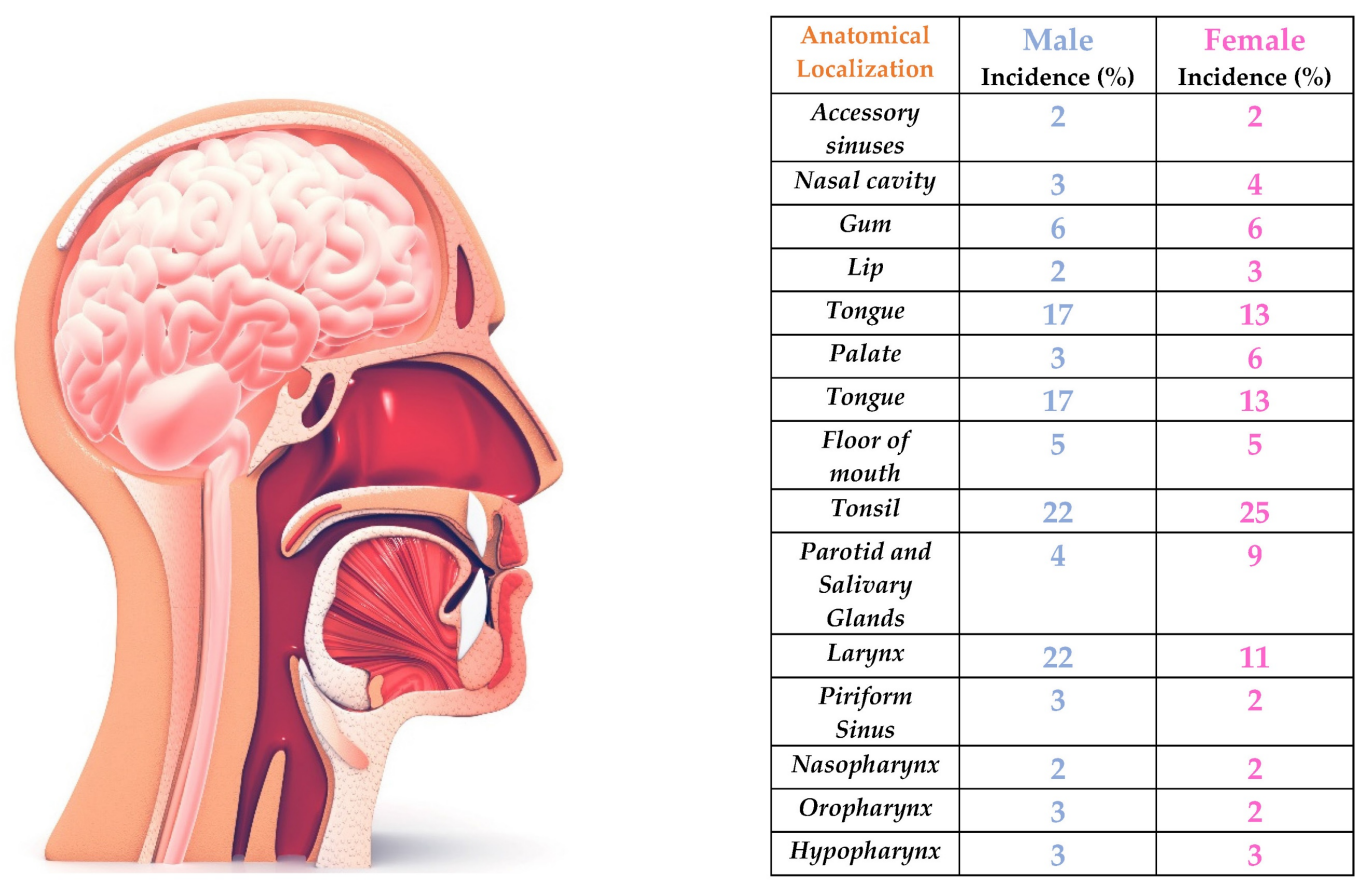
**Head and neck cancer statistical incidence.** HNC can appear in multiple anatomical districts: the most common are tonsils, followed by larynx for men and tongue for women.

Head and neck cancer (HNC) accounted for more than 66,920 new cases in 2023 (49,190 men and 17,730 women), causing more than estimated 15,400 deaths (11,210 men and 4,190 women), accounts this disease for about 4% of all cancers in the United States alone 1. Worldwide, the incidence of these heterogeneous types of cancer is equally high, with more than 562,328 people affected in 2020, being the 7th leading cause of deaths for all type of cancer 1,2. Head and neck cancer usually arise from the mucosal surfaces of head and neck region, but also in salivary glands. HNC includes cancers that develop in the oral cavity, larynx, nasal cavity, and salivary glands (Figure 1); the worldwide 5-year median survival rate at 50% of cases, with the hypopharynx experiencing the worst outcomes 2.

HNC is greatly impacted by environmental (human papilloma virus infection is a known risk condition) and genetic factors, but behavioral habits loom large: alcohol and/or tobacco consumption are present in more than 80% of the total HNC cases. Smoking alone accounts for 42% of cancer incidence 3-4; if both factors are present, the risk for oral and laryngeal cancer increase by 35-fold 5. Individual's statistics can only reaffirm the urgency to really challenge HNC: understanding their metabolic pathway and interaction can go a long way in helping general people's health. These data help us to understand the magnitude of the problem, and at the same time make us recognize the need to study in detail the emergence and development of HNC: being able to fully understand the molecular mechanisms will be of great help in devising effective strategies to deal with these pathologies. As usual in cancer patients, the clinical therapy includes surgery, chemotherapy and radiotherapy, and where possible, immunotherapy, also in combination with each other. If HNC is diagnosed in the early developmental stages (I or II), a benign course of the pathology can reasonably be assumed; conversely, if discovered in the late phases (III or IV), the degree of remission of the disease is significantly lowered 6.

HNC cancer etiology is a complex phenomenon, that can arise from different causes and different prognoses in several different districts; hence, it could be difficult to find a common base/perspective for this type of cancer; yet it is possible to find common ground in all these cancers and the internal and external factor that can trigger their development. Particularly intriguing is trying to elucidate the role of oxidative stress (OS) in promoting/facilitating the onset and development of head and neck tumors. As demonstrated by numerous researchers, HNSSC is characterized by high genetic and metabolic heterogeneity, and OS plays a central role in the emergence and development of these tumors. The presence of cellular OS enhances significantly - along with the reactive oxygen species (ROS) overabundance - the possibility of cancer arising.

## 2. Head and neck cancer metabolism and oxidative stress

As typical know in cancer cells, HNC characteristically shows exponentially, disproportionate, and unlimited proliferation. To support this growth, these cells adopt a peculiar metabolism, which promotes and enhances glucose uptake and anaerobiotic glycolysis, ultimately leading to Adenosine triphosphate (ATP) and lactate production, the latter producing the well-known Warburg effect 7. This choice has a profound effect on the overall cell metabolism, and conversely, on human body's ability to effectively counteract cancer development. Normally, cells prefer to degrade glucose through aerobic glycolysis, which produces pyruvate that enters the mitochondria in the Tricarboxylic Acid Cycle (TCA) cycle, and subsequently in the electron chain transport, which led to the production of 32 ATP molecules from a single glucose fraction 8. Conversely, during anaerobic glycolysis, the net ATP production is much lower, with only 2 molecules 8.

By adopting a very simplistic metabolism - i.e., bypassing the mitochondria and its aerobic metabolism - cancer cells can obtain the energy needed for their duplication, and at the same time infringe our ability to inhibit cancer cells specifically. In fact, by abiding by any kind of cellular differentiation - they mostly promote mitosis, i.e., cellular division -, it is difficult for our strategy against cancer to target any kinds of molecular switches, that are just not employed in the cancer cells 7,8.

The OS is an imbalance between the ROS presence and the antioxidants species at cellular level. There are several ways in which OS can impact cancer onset and development; by hypoxia regulation, but the aim of cancer development is to block the body's defense to spread the disease all over the tissue 9. The ROS are chemically reactive molecules derived from molecular oxygen that play significant roles in cellular signaling and homeostasis. ROS include superoxide anion (O2·-), hydroxyl radical (OH·), hydrogen peroxide (H2O2), and singlet oxygen (·1O2) 10. Under normal physiological conditions, ROS are produced as byproducts of cellular metabolism through processes such as mitochondrial respiration, enzymatic reactions, and immune responses 7-9. ROS function as signaling molecules involved in cellular processes such as proliferation, differentiation, and immune responses 9. An excess of pro-oxidant species production versus antioxidant species provokes OS: in fact, ROS excessive production could be the byproduct of pathological conditions, particularly of OS situations 8. For the oral cavity, one of the most important ROS sources is i.e., periodontal inflammation 10. Harmful habits such as cigarette smoke, and drug use, plays a prominent role in ROS production; also, diet - namely, ethanol intake - and high fat and/or high protein diet seems to favor ROS production 10. Also, dental treatment with laser light, ultraviolet light, ozone, etc., as well the materials used in dental practice, such as dental composites, can contribute to the ROS production 11.

## 3. Oxidative stress and its importance in HNC tumor development

**Figure 2:**
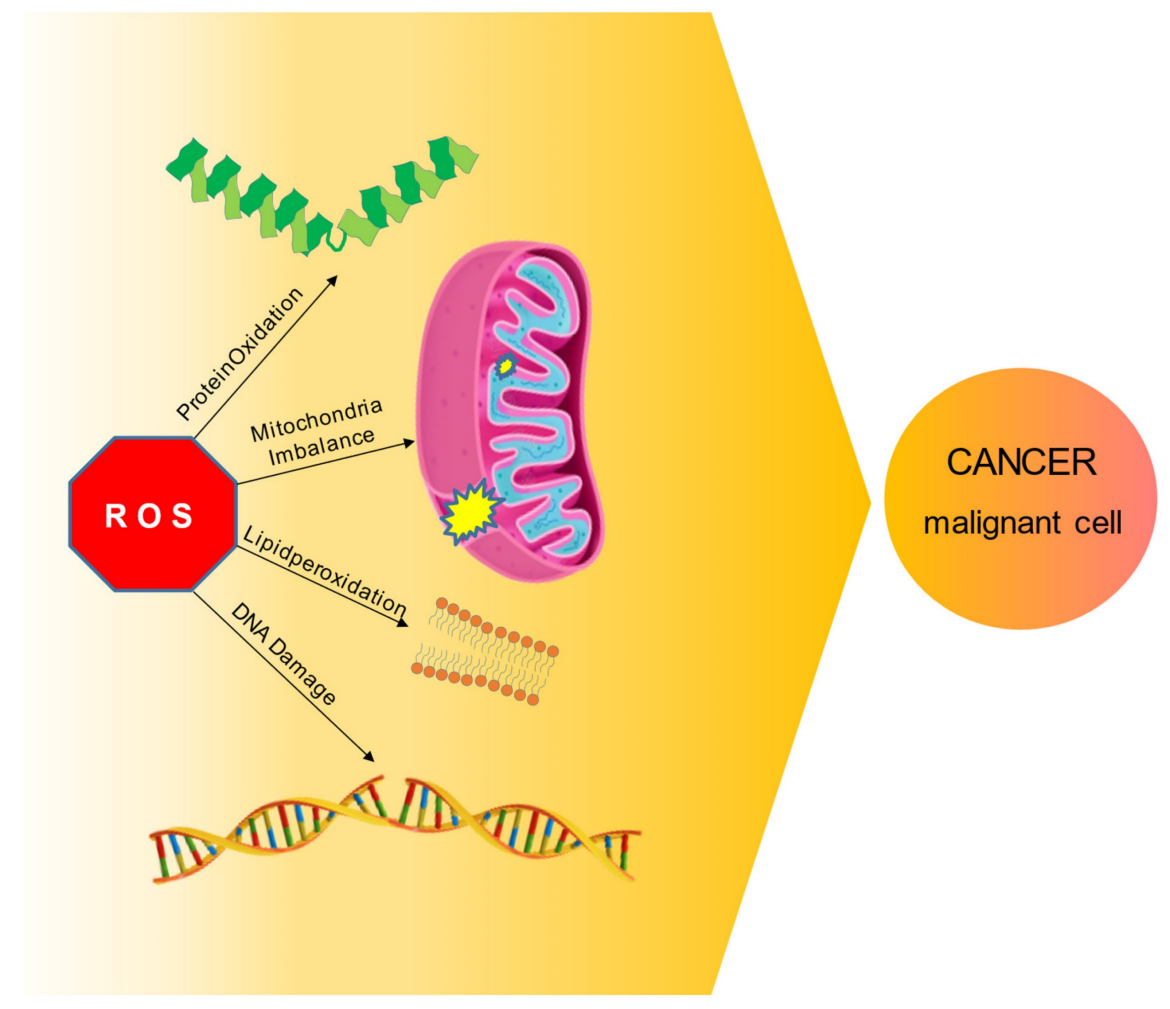
ROS molecular mechanism and HNC cancer. ROS overabundance in cell can have serious nefarious consequences, disrupting macromolecules and altering multiple biological pathways, ultimately leading to malignant cell transformation and cancer development.

To fully perform their normal biological and metabolic activity, cells need to effectively control ROS production. DNA maintenance, protein regulation, transcription factors activation, immunity system, energy metabolism, pathway control, cell growth, and differentiation, and ultimately cell division (in a controlled manner) or apoptosis, are all biological processes that can be negatively influenced by an excess of ROS 12. Is well know that ROS presence does not have to be considered only as a harmful effect: experimental research has extensively demonstrated that in physiological conditions ROS signaling as a second messenger in cells is crucial for a variety of biological actions, such as gene expression, signal transduction, and receptors activation 12-13, and also wound healing, tissue regeneration and protection from pathogens 14-15. However, if ROS levels increase above the usual threshold can lead to serious nefarious consequences for the cell: just the modification in the macromolecules - proteins oxidation, nucleic acids damage, carbohydrates, and lipid peroxidation - could render impossible a healthy cell life; hence, maintaining ROS levels at adequate levels becomes an essential necessity for the cell (Figure 2).

A major part of these molecules is a byproduct of oxidative metabolism, usually generated in the mitochondria. Oxygen is a crucial factor for human metabolism in general: oxidative phosphorylation, the role of multicopper oxidase enzyme (MCO) 16, arachidonic acid pathway - lipoxygenases (LOX), and cyclooxygenases (COX) -, and significantly, inflammatory pathway and particularly endothelial cells require molecular oxygen to be completed 17-19. Regulating ROS homeostasis is a key factor for cellular well-being: the lipid and carbohydrate peroxidation, protein oxidation, DNA damages and inflammation state due to ROS overproduction could facilitate the development of head and neck cancer 20. ROS can also cause an imbalance in mitochondrial metabolism, causing change in membrane permeability and halting the ATP production, which in turn cause cell cycle alterations, ultimately leading to the cancer appearance (Figure 2) 21. Cells normally cope with an excess of ROS through their scavenging capacity; however, in stressful conditions this ability falls short of matching the mitochondrial production of ROS 22-23.

The fight against cancer is truly an uphill battle: so much heterogeneity in the causes, and development and symptoms make it extremely complex and difficult to find a single way to counteract it. However, developing and fortifying our protective mechanisms against cancer insurgence - chemoprevention - can have a very long way in helping us against this powerful enemy. Introduced by Wattenberg 24, the concept of chemoprevention lies on the basis that several natural substances seem to display an ability to prevent cancer development. Interestingly, polyphenols molecules are a class of molecules that can have a positive effect on cancer onset and development, by affecting several distinct metabolic pathways, hence maybe having a synergistic effect: cell cycle, apoptosis, cell division, energy metabolism, DNA maintenance, are just some of the mechanisms of action of polyphenols in our metabolism 25-29. There is a clear relationship between polyphenol intake and its positive effect on our health: the scientific evidence is stunningly clear, regarding the onset and development of several different pathologies, from cardiovascular diseases, neurogenerative disorders, obesity, diabetes, inflammatory disease, aging, and of course many different types of cancer, including HNC 30-37.

## 4. Polyphenols: structure and function

**Figure 3:**
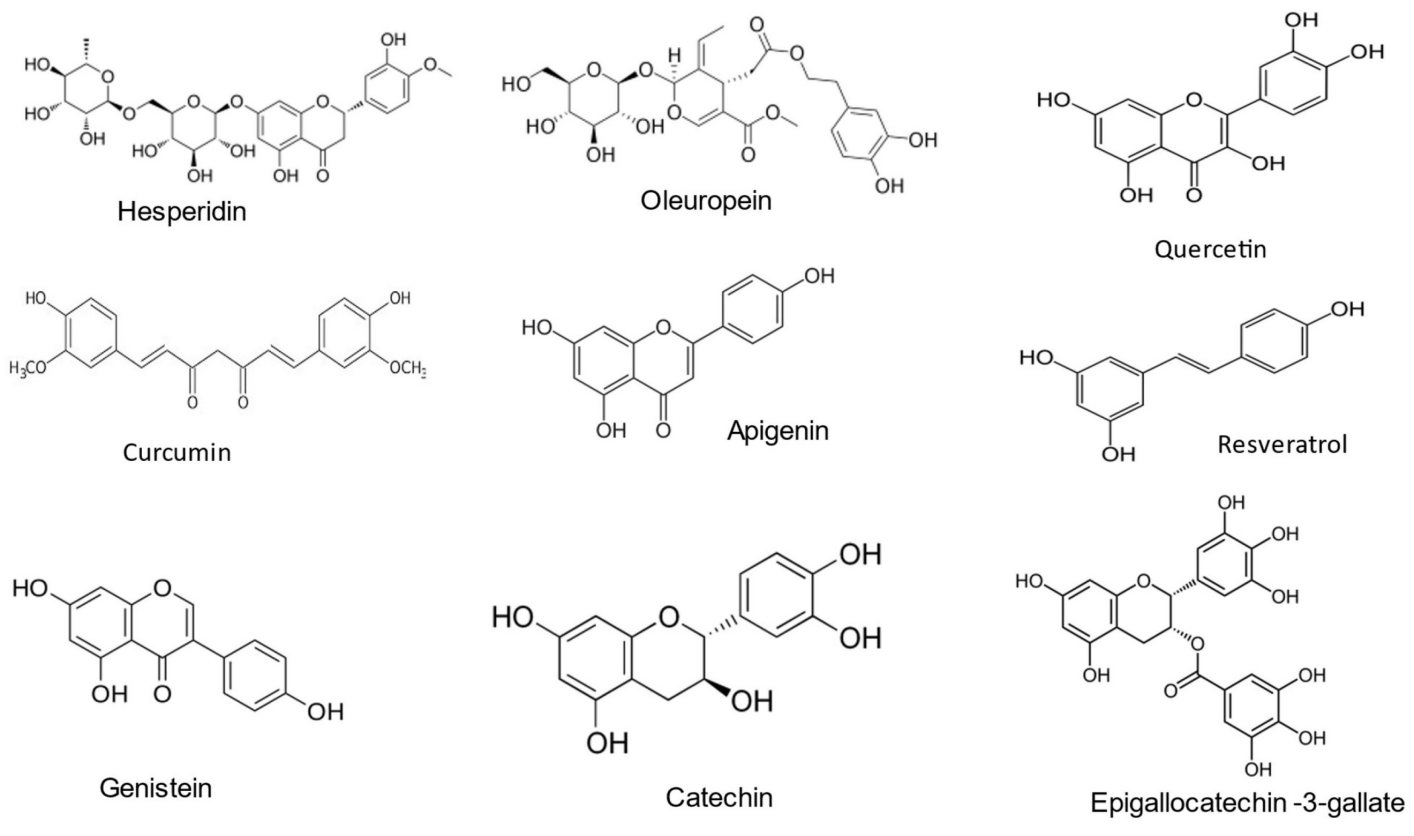
The chemical structure of different food polyphenols.

Polyphenols group encompass more than 10,000 molecules: their classification could be operated in several ways, for example by the number of their phenolic groups, or by dividing them into flavonoids and non-flavonoids; in any case, all the molecules classified as polyphenols possess at least one aromatic ring groups with one or more hydroxyl functional groups attached (Figure 3) 38-39. Polyphenols are classified into different subclasses, including flavonoids, phenolic acids, stilbenes, and lignans, each with unique chemical structures and properties 40.

The therapeutic potential of several different polyphenols molecules has been extensively studied: they are mainly present in the vegetables and fruits groups and seems totally conceivable that are at least partially responsible for the benefit of a plant-based diet. Flavonoids are present mainly in vegetables, cereals, fruits, and legumes. Quercetin is a ubiquitous flavonoid present in a large variety of fruits (apples, grapes, olives, citrus fruits, berries), vegetables (tomatoes, onions, broccoli, capers), beverages (tea and red wine), and herbal extracts; however, its concentration in all species is quite low 41. Ellagic acid is found in quercus species and particularly in pomegranates 42. Hesperidin, a flavanone glycoside, is the most abundant polyphenol in citrus fruits 43. Olive oil, a food often cited as an example as of source of good-for-health unsaturated fatty acids, contains a fair amount of hydroxytyrosol (HT) and oleuropein, polyphenols belonging to the catechol family 44. For a more complete list of foods with the highest polyphenol content, please refer to Perez-Jimenez et al. 45.

The main representatives of the catechin subfamily are epigallocatechin and epigallocatechin gallate (EGCG), which can be harvested from many types of herbs, fruits, legumes, and algae. Due to their relatively high content in berries, tea - especially green and white tea - 46, is also an important source of catechins 47. Resveratrol is one of the most studied molecules of the stilbene subfamily, which is usually found in grapes, and consequently in red wine 48. Therefore, due to their widespread distribution in fruits, cereals, legumes, and vegetables, and ultimately in all plant-based food, polyphenols are the ideal candidate to help the general population cope with oxidative stress situations: by adhering to the recommended guidelines, for 5 portions of fruit and vegetables per day, polyphenols intake ingested could contribute to the individual's health protection 49. However, it is impossible to correlate the intake of polyphenols present in a peculiar food and correctly evaluate the subsequent amount of polyphenols absorbed: besides environmental factors, such as light, temperature, water availability, nutrient status, and biotic stress, seasoning, cultivar, food processing - cooking, frying, toasting, etc. - can significantly affect polyphenol biosynthesis in plants. These factors modulate the expression of key biosynthetic genes and the accumulation of specific polyphenols, and ultimately affect our ability to estimate their bioavailability, which could render ineffective polyphenol's therapeutic potential in human metabolism. Hence, assessing an individual adequate dietary intake needs to consider polyphenol's bioavailability, which is different for every molecule, and is a very hard parameter to estimate: in fact, it is deeply affected by intestinal absorption, which can range from 3% of chlorogenic acid up to 43% of caffeic acid 50-51.

Usually, polyphenols in foods - except for the flavanols members - are in conjugated form (with carbohydrates), altering their solubility, digestion, and absorption properties, favoring their degradation and greatly limiting their absorption 52. At this stage, it is extremely important to underline the role played by the intestinal microbiota: the biotransformation reactions of polyphenols *in vivo*, - i.e., sulfoxide reductase, nitro reductase, glucuronosyltransferase - are all microbial enzymes 53-54 and, consequently, microbiota community composition can greatly influence their function 55.

## 5. Polyphenols antioxidant activities

**Figure 4:**
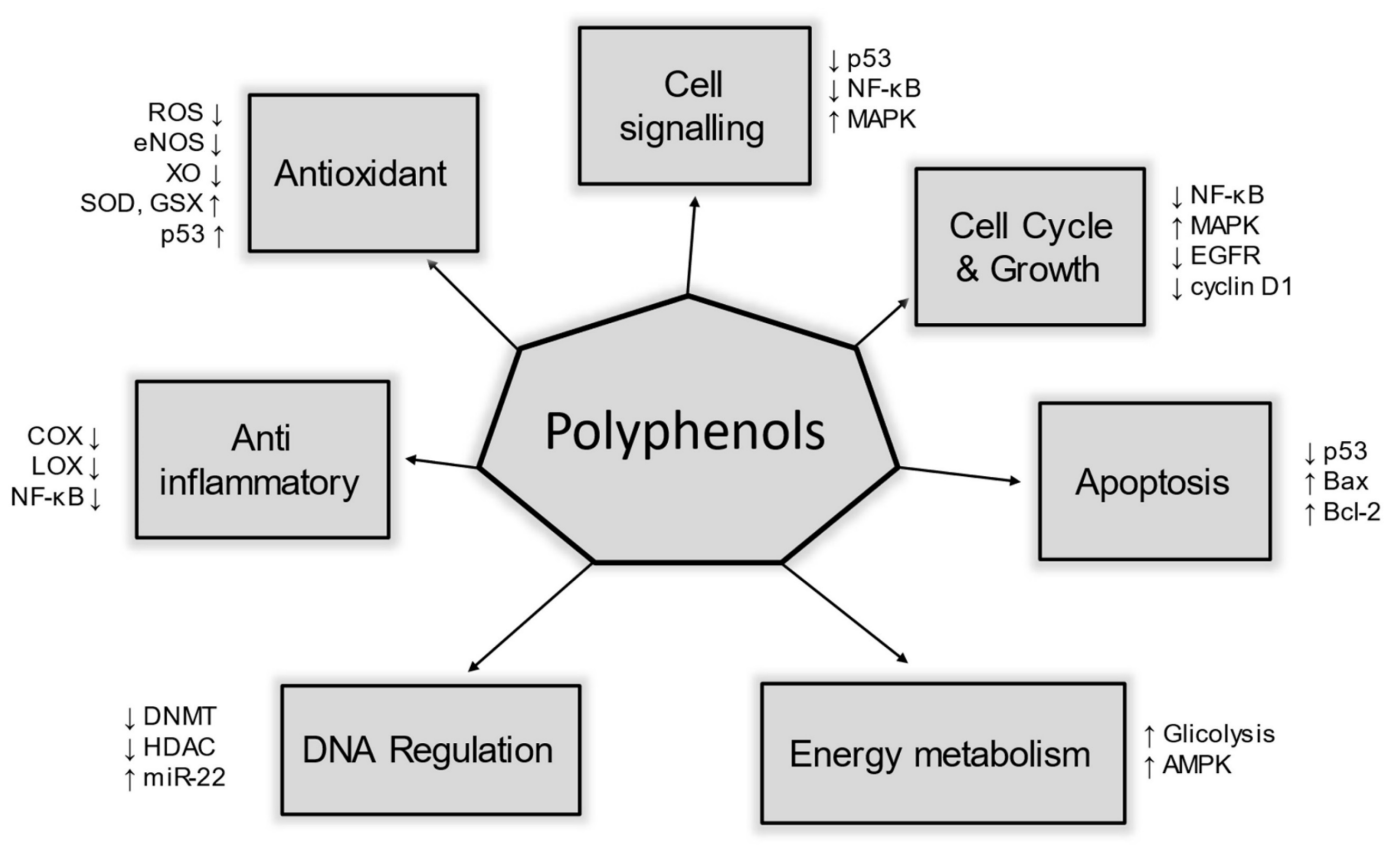
The role of polyphenols and human health. Dietary polyphenols can impact several proteins involved in many different aspects of our metabolism, including many pathways that are altered in HNC cancer cells.

The presence of antioxidant molecules in cells could be defined as a key factor regarding our health: scientific literature has extensively proved that they prevent DNA damage, including the mitochondrial genome, thus positively affecting mitochondria biogenesis 56. Polyphenols play a role in several molecular pathways that help us to manage oxidative stress. These pathways include enzymatic activity, metabolic regulation, membrane integrity, signal transduction, genetic activation, and epigenetic modifications. All of these are biological mechanisms involving polyphenols. In Figure 4, were illustrated some of the biological actions mediated by polyphenols in our metabolism.

By interacting with non-polar compounds found in the hydrophobic inner membrane layer, polyphenols support the proper functioning of membranes 57. This interaction helps preserve the rate of lipid and/or membrane protein oxidation. A study on hyperlipidemic rats demonstrated that the extract of Sempervivum tectorum exhibited antioxidant activity, protecting the organism against lipid peroxidation, and ultimately stabilizing the membranes 58. These molecular interactions provide insights into the polyphenol's beneficial effects.

The documented evidence shows that polyphenols can interact with endothelial nitric oxide synthases (eNOS). They can modulate nitric oxide production, signaling, and metabolism, thus regulating eNOS expression and activity 59. Oxidative stress can reduce nitric oxide bioavailability, which in turn contributes to endothelial dysfunction, a characteristic feature of cardiovascular disease. Bacterial infection can further worsen the oxidative injury by stimulating inducible NOS (iNOS) expression. However, polyphenols can improve macrophage function by inhibiting lipopolysaccharide-induced iNOS expression, thereby reducing oxidative stress 60. Some *in vitro* studies, demonstrated that the total polyphenols derived from Allium cepa possess the capability to inhibit the activity of xanthine oxidase (XO) 61. This antioxidant activity of flavanols is significant because XO activity has been associated with oxidative stress-related diseases, particularly ischemia 62, as the stimulation of XO can result in excessive production of free radicals.

Another important target for the antioxidant activity of polyphenols is NADPH oxidase (NOX). The NOX family comprises multiple members present in all human tissues and serves as one of the primary producers of ROS in various cells. In neutrophils and macrophages, NOX plays a crucial role in the oxidative burst, which involves the production of ROS for pathogen elimination 63. The polyphenol Curcumin, can modulate NOX activity, as observed in supplementation studies using mouse C2C12 myoblasts, where it directly inhibited NADPH oxidase 64. Additionally, it has been demonstrated that resveratrol could act as an O2*- scavenger, effectively and directly reducing ROS production mediated by NOX 65.

Advanced glycation end products (AGEs) are obviously stimulated by ROS, as well as the protein kinase C (PKC) pathways, leading to an activation of gluconeogenesis and lipogenesis. Experimental studies have demonstrated that polyphenols have the capability to inhibit SGLT1, thereby limiting the intestinal absorption of carbohydrates 66. Moreover, when PKC is overexpressed, it can exacerbate oxidative stress by stimulating NADPH-oxidases and lipoxygenases, which are enzymes known to generate ROS, as evidenced in human platelet suspensions 67.

The arachidonic acid pathway plays a crucial role in different diseases, including cancer development, arthritis, asthma, and general inflammatory processes 68. The breakdown of arachidonic acid through multiple enzymes, particularly COX and LOX, leads to the production of prostaglandins and leukotrienes, which are key factors in managing the inflammatory process. Polyphenol supplementation can influence the metabolism and pathway of arachidonic acid. For example, quercetin can modulate the activity of COX, LOX, phospholipase A2s (PLA2s), and cytochrome P450 (CYP) 68. Similarly, curcumin exhibits potent inhibition of arachidonic acid-induced inflammation *in vivo*. Therefore, the action of polyphenols ultimately reduces the progression of inflammation, playing a crucial role in preventing adverse health outcomes.

Oxidative stress encompasses many modifications that affect energy metabolism 69 and its regulation, as well as gene expression 70. It is not surprising that a wide range of genes, including ferritin 71-72, collagen 73, and transcription factors such as CREB 74 and STAT3 75, along with AMPK 76 and several proto-oncogenes 77, are transcriptional activated in response to increased cellular oxidation. Given their antioxidant capacity, polyphenols can contribute to individual antioxidant and anti-inflammatory defense through different mechanisms: a) by inhibiting the production of ROS and acting as scavengers of free radicals 78-80; b) by stimulating the production of prostaglandins and leukotrienes, which are anti-inflammatory molecules 81; c) by reducing levels of pro-inflammatory cytokines 82. The TNF-α, IL-6, and serum amyloid A, well-known inflammatory marker levels, are significantly reduced by using a blend of green tea polyphenols, comparable to the effects of sulfasalazine, the standard drug for patients with inflammatory bowel disease (IBD) 83.

Animal models with acute or chronic inflammation have been used to test several polyphenols molecules activity: kaempferol, resveratrol, HT, curcumin, and genistein have displayed anti-inflammatory activities in both animal models 84. Quercetin is beneficial for both chronic and acute inflammatory processes, while curcumin and green tea have been utilized in the treatment of stress-related neurodegenerative diseases 85-86. Polyphenols can potentially mitigate inflammatory processes through enzymatic and signaling systems, such as tyrosine and serine-threonine protein kinases, which regulate anti-inflammatory cell activation, growth, and differentiation (e.g., T cell proliferation, B lymphocyte activation), as well as cytokine production 87.

Moreover, polyphenols can induce the expression of antioxidant enzymes such as superoxide dismutase (SOD), catalase, and glutathione (GSH) peroxidase (Px) 88. This effect has been demonstrated in both *in vitro* and *in vivo* experiments using resveratrol, specifically in intestinal epithelial cells and porcine enterocytes isolated from the jejunum (IPEC-J2) 89.

## 6. The Overall polyphenols anticancer activities in HNC

The therapeutic use of polyphenols against cancer is based on their diverse range of biological activities, encompassing antioxidant effects, interactions with cellular receptors, apoptosis induction, modulation of cell signaling, alterations in cell cycle, regulation of cell proliferation, inhibition of angiogenesis, influence on inflammation and the immune system, epigenetic modifications, and modulation of gene expression 90. Polyphenols can also impact our health by influencing conditions such as diabetes, metabolic syndrome, hypertension, cardiovascular disease, and the production of metabolites by gut microbiota 91.

The scientific literature extensively deals with the potential anticancer activity of polyphenols on various types of cancer cells, including human colon cancer, lung cancer, breast cancer, ovarian cancer, and hepatocellular cell lines 92-96. Polyphenol extracts have demonstrated effectiveness in preventing skin cancer and have shown utility in the treatment of this highly aggressive form of cutaneous cancer 97. A study conducted on HNC revealed significant growth inhibition when a blend of polyphenols (quercetin, curcumin, green tea, and resveratrol) was employed 98.

Dietary phytochemicals can display their anticancer potential through different molecular mechanisms; it is evident that polyphenols can interact with and modulate different signaling pathways involved in the onset and development of cancer. Therefore, the development of anti-cancer therapies based on the utilization of polyphenols holds promising potential for cancer treatment 99.

The epidermal growth factor receptor (EGFR), is deeply involved in HNC onset and development: according to the recent statistics, more than 90% of all HNC display an EGFR overexpression; moreover, high EGFR levels are inversely correlated with poor prognosis and cancer's patient survival 100. EGFR belongs to the ErbB family, which include four members (ErbB1-4): many different polyphenols molecules possess well-documented abilities to exert their influence upon members of the ErbB receptor family in various cancer cell types. Oleuropein and HT emerge as transformative agents capable of degrading EGFR in several cancer cell lines, as well as quercetin, apigenin, EGCG, and resveratrol. Curcumin indeed surpasses the effectiveness of the gefitinib drug in inhibiting colon cancer cell growth, positioning it as a potent agent for suppressing tumor proliferation 101.

Distinguishing between different signaling pathways can be a challenging task as many of them regulate similar cellular processes, such as cell growth, differentiation, and proliferation, albeit with different involved proteins. Thus, attempting to elucidate the role of each pathway could be debatable. Moreover, these pathways can interact and exhibit crosstalk. For instance, the nuclear factor-kappaB (NF-κB), and Hedgehog signaling play crucial roles in determining cellular neoplastic transformation 102-103.

### 6.1. Polyphenols and HH/GLI pathway

The Hedgehog pathway (HH) serves as a critical regulator of cell differentiation and growth, particularly during embryonic stages. Recent studies have highlighted an overactivation of this pathway in several human cancers 104. The cascade pathway is governed by the interaction of three components: the sonic hedgehog (Shh) ligand, its pathway repressor Patched 1 (Ptch1), and the pathway activator smoothened (Smo), a transmembrane G-protein. Normally, Smo is negatively regulated by Ptch1. Unlike other signaling pathways, HH typically operates under negative regulation. When Shh binds to Ptch1, it activates Smo, leading to the nuclear translocation of GL1, a zinc finger transcriptionally repressive factor, resulting in cellular proliferation 105. Dysregulation of the HH signaling pathway has been observed as a clinical hallmark in the development and progression of various cancer types, including gastrointestinal, prostate, lung, breast, and brain tumors 106-110.

Several different polyphenols molecules have individually demonstrated the ability to regulate the HH/GLI pathway. Curcumin induces cell cycle arrest and apoptosis through the HH signaling pathway by inhibiting the transcriptional activity of GL1. Genistein can block MCF-7 breast cancer cells by inhibition of Sonic hedgehog activity 111, and apigenin impact HH/GLI pathway in malignant mesothelioma mouse cell 112. EGCG halt the growth and metastasis of human chondrosarcoma cells. In liver of cancer-induced mice, oral administration of EGCG significantly reduces the expression of Smo and GL1 113. Resveratrol effectively suppresses hypoxia-induced HH stimulation in pancreatic cancer cells 114. Both EGCG and theaflavin can effectively counteract the carcinogenic effects of N-nitrosodiethylamine (NDEA) in mice by inhibiting the catalytic transformation of PTCH1, thereby preventing the activation of the HH signaling pathway 115.

### 6.2 NF-κB pathway modulation

The NF-κB plays a role in different processes, including inflammation, immunoregulation, apoptosis, cell growth, and proliferation. NF-κB is a family of transcription factors consisting of several members, such as NF-κB2 p52, NF-κB1 p50, c-Rel, RelA/p65, and RelB 116. These proteins can form homo/heterodimers and bind to specific DNA sequences known as their target sites. All family members possess a conserved amino acid domain called the Rel Homology Domain (RHD), which is essential for dimerization, inhibitors binding (IkB), nuclear translocation, and DNA binding.

Normally, NF-κB is bound to its inhibitor IkB, thus remaining confined to the cytoplasm: to be activated NF-κB is phosphorylated by the IκB kinase (IKK) complex, which leads to the IkB degradation and allows the transcription factor translocation into the nucleus 117. Once in the nucleus, NF-κB regulates gene expression, activating different genes depending on its composition, with many involved in inflammation, cell growth, and differentiation. Supplementation with green tea polyphenols can inhibit mitogen-activated protein kinase (MAPK or MAP kinase) signaling in human umbilical vein endothelial cells (HUVECs) that can participate in the regulation of NF-κB transcriptional activity 118. Catechins were able to suppress NF-κB signaling in a rat model by preventing NF-κB nucleus translocation 119; also apigenin and genistein blocked NF-κB interaction with DNA targeting sequence in a murine model 120.

### 6.3 Polyphenol, cell growth arrest and apoptosis

Apoptosis, a programmed cell death, is a defense mechanism activated when cells are damaged beyond repair, preserving the organism from aging, infection, or other degenerative disease 121. This genetically regulated process is particularly crucial in countering cancer, which is characterized by uncontrolled cell division: indeed, several anticancer drugs induce apoptosis, an essential feature to prevent the development of neoplastic conditions. The apoptotic process involves distinct pathways with different protein, and can be triggered either by intrinsic factors, such as extensive DNA damage, ischemia, oxidative stress, or infections, or by extrinsic factors, which involve the interaction of specific membrane receptors with pro-apoptotic molecules produced elsewhere. Both intrinsic and extrinsic pathways require the activation of the proteolytic caspase cascade, ultimately dismantling and eliminating the dying cell 122.

Olive oil is widely recognized for its antioxidant properties but can also be used against cancer. Several studies have shown that oleuropein decreases cancer cell viability and exhibits pro-apoptotic effects through the p53-dependent pathway and by activating BAX and Bcl-2 genes in breast cancer cells (MCF-7) 123. HT can also influence cell cycle progression by arresting cancer cells in the G0/G1 phase and reducing cyclin D1 levels.

In ovarian cancer cells curcumin - in a p53-independent way - can induce apoptosis through the activation of p38 kinase, downregulation of Bcl-2 expression, and modulation of Akt signaling 124. In the MOLT-4 human leukemia cell line, quercetin interacts with the PI3K-dependent/AKT pathway, leading to a decrease in mammalian target of rapamycin (mTOR) activity. Consequently, the anti-apoptotic protein Bcl-2 is downregulated in cancer cells 125.

Synergistic effects of polyphenols have been observed in various biological processes. Curcumin can induce apoptosis in pancreatic cancer cells line, both *in vitro* and *in vivo* 126. Quercetin and ellagic acid demonstrate synergistic effects in p53 phosphorylation, stimulating BAX expression and translocation of p53 protein into mitochondria, ultimately resulting in pro-apoptotic effects. Similar synergistic effects on p53 phosphorylation were observed when treating human lung cancer cells with isoflavones and curcumin, leading to decreased cell growth and proliferation 127. Resveratrol exhibits potential effectiveness in preventing cancer development in several cancer cell lines, including prostate, breast, stomach, colon, lung, intestinal, thyroid, and pancreatic cancers 125. Resveratrol induces apoptosis in human leukemic cells by decreasing Akt activation through Ras downregulation 128.

Although resveratrol shows promising anti-cancer potential, its efficacy has been limited to tumors with direct contact, such as skin cancers or gastrointestinal tract cancers, rather than human solid tumor cells, due to its poor bioavailability 128.

### 6.4 Polyphenols modulation of p53

Approximately half of all human tumors display an altered functioning of p53, a crucial regulator in multiple human metabolic pathways 129. In fact, p53 plays a vital role by regulating DNA maintenance and repair, halting cell cycle progression to assess DNA damage, and initiates apoptosis. More than 100 genes have been identified as targets of p53, encompassing various aspects of cellular metabolism. Post-translational modifications such as acetylation, methylation, phosphorylation, and ubiquitination can modulate p53 activity in response to a wide range of stresses 130.

Therefore, it is not surprising that p53 can be regulated through various mechanisms, either positively or negatively, involving different pathways and proteins. One common pathway involved in p53 inhibition is the direct interaction between p53 and the Mouse double minute 2 homolog (MDM2). The MDM2 carries out its activity through several mechanisms: 1) facilitating proteasome-mediated degradation of p53; 2) preventing p53 from binding to its DNA target sequence; and 3) promoting the export of p53 out of the nucleus 131. Experimental evidence suggests that polyphenols can bind to MDM2 through stable hydrophobic interactions, preventing the inactivation of p53 132.

EGCG, resveratrol, curcumin, genistein, and quercetin can upregulate p53 expression, inhibit cell growth and proliferation in several human cancer cell lines by decreasing cyclins D1 and D2, increasing p21 and BAX synthesis, and triggering apoptosis 133-134. Specifically, EGCG can directly regulate p53 by stimulating its phosphorylation and acetylation, leading to enhanced stability and activity of p53 135. Besides, p53 can be targeted by the theaflavin's biological activity, leading to positively telomerase regulation through inhibition of Telomerase reverse transcriptase (hTERT), a critical factor for cell life expectancy 136. Moreover, theaflavin - through p53 pathways - can downregulate glycolysis and angiogenesis - suppressing vascular-endothelial growth factor (VEGF) expression -, promoting apoptosis through Bcl-2 inhibition 137.

Numerous studies have suggested that resveratrol can triggers apoptosis in cancer cell in a p53-dependent manner, via MAPK activation 138. Therefore, polyphenols administration can modulate p53, regulating various aspects of cancer progression, including initiation, proliferation, survival, migration, angiogenesis, and metastasis. Importantly, the combination of polyphenols with chemotherapy or radiotherapy can synergistically upregulate p53 139.

### 6.5 Epigenetic and DNA modification

Preventing cancer initiation and progression often could significate preserve the stability and integrity of our genome: in this regard, epigenetic regulation and gene silencing/activation plays a pivotal role for cell development and differentiation. DNA methylation and chromatin modifications, particularly histone acetylation, are crucial for proper development and differentiation, but dysregulation can lead to severe human pathologies, cancer included. The tumor microenvironment acts as an amplifier for epigenetic modifications, facilitating early and frequent remodeling of DNA functionality, and promoting cancerous transformation 140. Targeting epigenetic modifiers could be a promising strategy for anticancer activity, given the potential reversibility of these changes.

Recent studies have suggested that polyphenols may have the ability to regulate epigenetic processes, which holds significant clinical and therapeutic implications 141. In ER-positive MCF-7 breast cancer cells, a combination of resveratrol and vitamin D can downregulate DNA methyltransferases (DNMT), leading to reduced promoter methylation of the phosphatase and tensin homolog (PTEN) gene and enabling protein transcription 142. Resveratrol treatment in breast cancer cells has been associated with DNA hypomethylation based on a genome-wide survey 143. Interestingly, combining resveratrol with another polyphenol, pterostilbene, reduces methylation at the ERa gene promoter 144.

Undoubtedly, dietary bioactive compounds like polyphenols can act as epigenetic modifiers, establishing a direct link between food and epigenetics, thereby presenting intriguing new therapeutic possibilities.

### 6.6 miRNAs modulation in HNC

MicroRNAs (miRNAs) are a class of small, single-stranded non-coding RNAs that plays a crucial role in post-transcriptional control: it is estimated that they regulate the expression of at least 30% of mammalian genes 145. Through binding to target mRNAs, miRNAs can downregulate gene expression, effectively regulating several cellular processes, including cell differentiation, growth, proliferation, and apoptosis; alterations in miRNA expression are considered critical in cancer initiation and development 146.

Curcumin ability to modulate miRNAs in cancer cells has been recently demonstrated: after treating pancreatic cancer cells with curcumin (10 μM), significant changes in the expression of 29 miRNAs were observed, with 11 miRNAs upregulated and 18 miRNAs downregulated 147. One of the miRNAs affected by curcumin treatment was miR-22, known for its tumor-suppressive function. Curcumin-induced upregulation of miR-22 effectively inhibited its target genes. These findings highlight the modulation of miRNAs by curcumin as an important mechanism underlying its biological effects. Furthermore, curcumin has been shown to induce DNA hypomethylation and inhibit several oncogenes, including histone-modifying proteins, in hepatocellular carcinoma cells 148.

Resveratrol and genistein are also capable of reducing carcinogenesis through the modulation of miRNAs. Additionally, EGCG not only impacts DNA methylation but also histone acetylation, influencing the enzymatic activities of histone deacetylases (HDACs). This EGCG capacity may explain its chemopreventive effect, as it modulates inflammation in cancer cells.

## 7. Polyphenols Therapeutic Potential in HNC: Epidemiological and Clinical Studies

Many epidemiological studies suggest that diets particularly rich in fruits and vegetables have cancer preventive properties 149-151. Polyphenols are deemed responsible, at least in part, for these beneficial effects, thanks to their anticancer activity both in animal and human models 152-153.

So far, very few studies have tested polyphenols administration in HNC. Searching the “Clinicaltrials.gov” database (last search: 5 August 2023), using the terms [head and neck cancer] and [polyphenols report], only 5 studies evaluate the use of polyphenols in HNC (Table 1).

Instead, are enlisted 30 clinical studies involving the administration of polyphenols in various types of cancer and 50 clinical studies using polyphenols to counteract oxidative stress (Table 2 and Table 3, respectively).

Given these premises, it seems counterintuitive to not try the use of polyphenols in HNC treatment and prevention. A high intake of polyphenols was linked to a nearly 50% decrease in gastric cancer 154. The consumption of stilbenes was shown to lower the risk of colorectal adenomas, while anthocyanin and flavanols were associated with a reduced risk of colorectal cancer 155. In a separate study focused on prostate cancer, the consumption of polyphenols was found to significantly decrease the risk 156, and isoflavones and flavones were inversely associated with the risk of bladder cancer 157.

Polyphenols intake was inversely associated with colon cancer in men, accordingly to the European Prospective Investigation into Cancer and Nutrition (EPIC) study, with a cohort of nearly half a million people from 10 different Countries 158. A study performed in Hong Kong with people who habitually consume green tea had a reduced risk of cancer of prostate cancer 159.

Although the use of polyphenols to combat oxidative stress or cancer appear extremely promising, the preliminary results of epidemiological studies must be evaluated with caution and extreme care. Ultimately, research of this type can give us some indications, but they do not provide us with any explanation or even a direct link between the use of polyphenols and pathologies. In fact, sometimes, if analyzed carefully, their results can be inconclusive: in the same work cited above, in some types of cancer, therapy with polyphenols did not change the incidence of the disease, or in some cases, it even worsened 158.

However, polyphenols can certainly be useful as nutraceutical coadjuvants: they have already demonstrated great therapeutic potential by increasing the effectiveness of traditional drugs. Furthermore, being molecules normally present in plants, they do not present any toxicity problems; indeed, their use seems to attenuate these side effects, which are present in almost 40% of all HNC patients 160.

## 8. Discussion

HNC is increasing worldwide, becoming more and more a challenging clinical problem. It is necessary to find viable strategy to contain this type of cancer, and moreover develop alternatives for prevention. As mentioned previously, incorporating an adequate amount of polyphenols into our diet or considering polyphenol supplementation can be highly beneficial for maintaining our health, preventing and helping our body to fight against oxidative stress 161. The abundant presence of polyphenols in plant-based foods justifies the general recommendation to consume ample amounts of fruits and vegetables for maintaining our well-being. Through *in vitro* and *in vivo* experiments, it has been clearly demonstrated that polyphenols can modulate numerous biological pathways. By addressing oxidative stress through diverse molecular mechanisms, their efficacy can be enhanced, opening the door for synergistic interactions among different polyphenol molecules 109.

It is important to recognize that each situation presents its own unique challenges and therefore requires a tailored approach. The ability to utilize different polyphenol molecules based on specific circumstances is a valuable asset. Furthermore, the effectiveness of combining different polyphenols has already been demonstrated in several experiments, highlighting the benefits of synergy. Notably, the use of polyphenols does not entail any side effects, as they are completely non-toxic. This aspect greatly enhances the utility of polyphenols in clinical therapy 37,162.

In the past two decades, significant advancements have been made in the field of food and nutritional sciences. Conventional nutritional recommendations, which primarily focused on the sufficient intake of nutrients to prevent disease development, have been superseded by the concept of personalized nutrition. This approach aims to optimize bodily functions and promote human health through the utilization of bioactive compounds such as polyphenols 148.

Different polyphenols molecules often act in different ways in our metabolism; hence, different polyphenols may act together to produce synergistic effects with enhanced health benefits. Understanding these synergistic interactions and exploring combinatorial approaches can lead to optimized therapeutic strategies. Overcoming the challenge of achieving the biological effectiveness of polyphenol supplementation in our bodies remains a major obstacle. In most cases, these compounds have low bioavailability and solubility, making it extremely challenging to accurately assess their true activity. It is crucial to develop new delivery methods and synthetic analogs that can enhance this critical aspect: although there are already some, further clinical trials are needed to investigate the bioavailability of polyphenols with innovative methods, like enriched foods containing free or encapsulated polyphenols 100.

Many studies on polyphenols primarily consist of observational, *in vitro*, or *in vivo* research. While these studies are valuable in elucidating the molecular mechanisms involved, clinical trials participating large cohorts are necessary to truly evaluate the effectiveness of polyphenol supplementation for human health. Without these clinical confirmations, the administration of polyphenols for therapeutic applications becomes challenging, as there is a lack of clear indications regarding their efficacy, optimal intake, and specific pathologies in which they can be utilized 161. Intriguingly, the results of a recent study investigated the potential anticancer effects of combining two natural dietary compounds green tea EGCG and resveratrol; these compounds were tested both *in vitro* (in cell cultures) and *in vivo* (in live animals) in the context of HNC 163. Overall, the study results suggests that the combination of EGCG and resveratrol, even at low doses where each compound alone has marginal effects on apoptosis, can synergistically enhance apoptosis and inhibit the growth of head and neck tumors. Besides, therapeutic strategy using also depends on the activation of the immune system against tumor 164-166; intriguingly, a combination of natural polyphenols, containing curcumin (C) with resveratrol (R) and epicatechin gallate (E), termed TriCurin, presenting a unique and synergistic molar ratio, seems to be highly efficient in stimulating the immune system against cancer cells and can be used as a safe immunotherapeutic agent to turn the immune system against HPV+ tumors 167.

However, the polyphenols therapeutic potential is becoming more and more evident, enticing the worldwide researchers with a landscape of therapeutic possibilities to uncover the precise and effective biological role of this molecules in counteract oxidative stress, enabling their implementation in preventive healthcare practices, according to the evidence-based dentistry 168.

## 9. Conclusion

Cancers of the head and neck are clearly socioeconomically patterned, with those from the poorest backgrounds having the greatest burden, but this socioeconomic risk is not entirely explained by smoking alcohol and dietary behaviors.

Polyphenols have the potential to be a strategic tool in the battle against cancer for several reasons. As a diverse family of molecules, they can combat cancer development through multiple mechanisms, increasing the likelihood of success. The idea of preventing or curing cancer through a polyphenol-rich diet, primarily sourced from plant-based foods, almost feels like a dream come true. Additionally, adopting healthy dietary habits can deter us from consuming comfort foods high in saturated fats and calories, which are detrimental to our health and may contribute to cancer incidence and progression.

Recent studies have demonstrated that dietary polyphenols could regulate several molecular pathways involved in cancer promotion and progression, suggesting chemopreventive and therapeutic capacity of dietary polyphenols against HNC.

However, the concentration, absorption, bioavailability, and pharmacokinetics of polyphenols can pose challenges to their beneficial effects against neoplastic diseases. Further research is necessary to fully understand the cellular mechanisms by which polyphenols operate, enabling the integration of these natural compounds into our cancer-fighting strategies. While overall results appear promising, they remain inconclusive. Randomized controlled clinical trials and meta-analyses are required to test the actual efficacy of polyphenols, providing continuity and perspective to *in vitro* and cell culture studies. Discovering the optimal combination of polyphenols for HNC, sustained by innovative delivery methods like liposomes and nanoparticles, could significantly benefit patients fighting this type of cancer worldwide.

## Figures and Tables

**Table 1:** Clinical trials with polyphenol supplementation in head and neck cancer patients.

HNC	NCT	Polyphenol Supplementation	Clinical Relevance
Oral leukoplakia	NCT00176566	Lozenge intake (green tea preparation)	A Phase II Trial to Assess the Effects of Green Tea in Oral Leukoplakia
Oral cancer - gum disease	NCT01514552	Strawberry gummy and placebo control	Use of Functional Confections in Promoting Oral Health
Carcinoma, Squamous Cell	NCT01496521	Drug: aspirin (100mg 6 months); Dietary Supplement: Tea polyphenols (300mg 6 months)	Chemoprevention of Esophageal Squamous Cell Carcinoma (ESCC) with aspirin and tea polyphenols
Gastric and Esophageal Cancer	NCT04027088	Dietary supplement: arginine, omega 3, olive oil polyphenols, carnitine and antioxidants	Effect of preoperative immunonutrition in upper digestive tract
Premalignant lesions of HNC	NCT01116336	Green tea polyphenon E	Phase I chemoprevention trial with green tea polyphenon e & erlotinib in patients with premalignant lesions of HNC

**Table 2:** Selected clinical trials with polyphenol supplementation in cancer disease.

Conditions	NCT	Polyphenol	Clinical Relevance
Recurrent Prostate Cancer	NCT01912820	Quercetin	Effect of Quercetin in prostate tissue from patients with prostate cancer
Prostate Cancer	NCT00685516	green tea, decaffeinated black tea	Green Tea, Black Tea in treating patients with prostate cancer undergoing surgery
Prostate Cancer	NCT00676780	Drug: Polyphenon E (EGCG)	Green tea extract and prostate cancer
Colorectal Cancer	NCT02439580	Annona muricata extract	Effect of A. Muricata leaves on colorectal cancer patients and colorectal cancer cells
Incident Breast Cancer	NCT00949923	Dietary Supplement: tea capsula	Green Tea in Breast Cancer Patients
Cervical Cancer	NCT03994055	Omega-3 fatty acids, Probiotics Antioxidants Soluble fiber	Effect of an Anti-inflammatory Diet on Patients with Cervical Cancer
Interstitial Pneumonia Neoplasms Malignant	NCT05758571	Drug: EGCG	Oxygen atomizing inhalation of EGCG in the treatment interstitial pneumonia in cancer patients
Advanced Lung Cancer	NCT03751592	Drug: Chlorogenic acid	Phase Ib/IIa Studies of Chlorogenic acid for injection for safety and efficacy of advanced lung cancer
Skin Cancer	NCT01032031	Dietary Supplement: Green tea + vitamin C high dose	The Effect of Green Tea and Vitamin C on Skin Health
Non-small Cell Lung Cancer	NCT01426620	Dietary Supplement: Blueberry powder	Standard chemotherapy with blueberry powder in nonsmall cell lung cancer
Colorectal Serrated Adenomas	NCT01360320	Dietary Supplement: Green tea extract of Camellia Sinensis	Minimizing the risk of metachronous adenomas of the colorectum with green tea extract -MIRACLE-study

**Table 3:** Selected clinical trials with polyphenol supplementation in oxidative stress conditions.

Conditions	NCT	Polyphenol	Clinical Relevance
CV disease and oxidative stress	NCT01541826	Chokeberry extract (250mgx2 for 12 weeks)	Study of Chokeberry to Reduce Cardiovascular Disease Risk in Former Smokers
Oxidative stress and inflammation	NCT01780922	Cranberry extract beverage	Effect of a Dose of Cranberry Beverage on Inflammation and Oxidative Stress
Oxidative stress and insulin resistant	NCT02479035	Red raspberry meal	Raspberries on Insulin Action and Oxidative Stress
Cardiomyopathy and oxidative stress	NCT01102140	POMx pomegranate polyphenol extract	The Impact of Pomegranate Extract on Chronic Cardiomyopathy Complicated by Renal Insufficiency (ImPrOVE): a Pilot Study
Metabolic syndrome, oxidative stress, systemic inflammation	NCT03265184	Green coffee extract	Green Coffee Extract Supplementation and Oxidative Stress, Systemic and Vascular Inflammation
Oxidative stress, inflammation	NCT02494739	Yogurt enriched with polyphenols	Antioxidant and Anti-inflammatory Effects of Yogurt Enriched With Polyphenols
Vascular oxidative stress	NCT03053986	Apple polyphenols	Effect of Apple Polyphenols on Vascular Oxidative Stress and Endothelium Function Study (APP trial_2016)
Oxidative stress in diabetic patients	NCT00682149	PomGT (0.5 g pomegranate extracts, 0.3 g green tea and 60 mg vit. C)	Effects of Polyphenol Containing Antioxidants on Oxidative Stress in Diabetic Patients
Oxidative stress	NCT00721643	Angel's plant - dark green leafy vegetable	Absorption Kinetics of Polyphenols in Angel's Plant (Angelica Keiskei)
Oxidative stress, exercise recovery	NCT04959006	Antioxidant supplement	Investigating a Natural Antioxidant Food Product on Oxidative Stress in Recreationally Active Participants
Maternal and fetal oxidative stress	NCT01584323	Pomegranate pills	Pomegranate to Improve Outcome in Pregnancies Complicated With Preterm Premature Rupture of the Membranes
Oxidative stress, gestational diabetes	NCT05393843	Omega-3 fatty acids, anthocyanins and alpha-cyclodextrins	Prevention of Maternal and Fetal Metabolic Complications With Diet and Nutraceutical Supplementation in Pregnant Women Affected by Gestational Diabetes: a Randomized, Double-blind Placebo Controlled Trial.
Oxidative stress	NCT03186573	Grape juice	Effect of Grape Juice Consumption on the Parameters of Oxidative Stress and Muscle Fatigue in Judo Athletes
Oxidative stress, cardiometabolic risk	NCT05771571	Olive oil, plus orange peel extract	Investigation of the Acute Effect of Novel Olive Oil on Postprandial Oxidative Stress Biomarkers (BioliveCT)
Oxidative stress, CV disease, inflammation,	NCT01674231	Grape (freeze-dried whole grape powder)	The Effects Grapes on Health Indices
Chronic obstructive pulmonary disease, oxidative stress	NCT03989271	Quercetin	Biological Effects of Quercetin in COPD
Oxidative Stress, CV Diseases	NCT02295878	Dietary Supplement: capsule containing seaweed extract	The Effect of Seaweed Derived Polyphenols on Inflammation and Oxidative Stress in Vivo - The SWAFAX Study
Oxidative Stress, CV Diseases	NCT04061070	Supplementation with threalose + polyphenols	Effects of Trehalose & Polyphenols in Vasculopathic Patients
			
